# Serosurvey for selected pathogens in Iberian roe deer

**DOI:** 10.1186/1746-6148-6-51

**Published:** 2010-11-16

**Authors:** Mariana Boadella, Tania Carta, Álvaro Oleaga, Gerardo Pajares, Marta Muñoz, Christian Gortázar

**Affiliations:** 1 IREC (CSIC-UCLM-JCCM), Ronda de Toledo s/n, 13071 Ciudad Real, Spain; 2 Asociación del Corzo Español (UCM), Madrid, Spain; 3 Conselleria do Medio Rural, Xunta de Galicia, Santiago de Compostela, Spain; 4 SERIDA, Servicio Regional de Investigación y Desarrollo Agroalimentario, Laboratorio de Sanidad Animal, Gijón, Spain

## Abstract

**Background:**

The roe deer is the most abundant and widespread wild Eurasian cervid. Its populations are expanding and increasingly in contact with livestock. This may affect the distribution of infectious diseases shared with other wild and domestic ungulates.

**Methods:**

We investigated the antibody seroprevalence against Pestivirus, Herpesvirus, Bluetongue (BT) virus, *M. avium paratuberculosis *(MAP), and *Brucella *sp. in 519 roe deer from different regions in Spain, south-western Europe.

**Results:**

No antibodies were detected against BT and *Brucella *sp. However, antibodies were detected against Pestivirus (1.5%), Herpesvirus (0.2%) and MAP (9.2%). MAP antibodies were detected in seven of the eight populations (range 5-16.4%).

**Conclusions:**

The detection of MAP antibodies in samples from most roe deer populations suggests that contact with MAP is widespread in this wildlife species. The highest prevalence was detected in sites with abundant dairy cattle and frequent use of liquid manure on pastures. Considering the results obtained regarding exposure to different pathogens, we suggest that antibody prevalences in this non-gregarious browser are largely determined by environmental factors, potentially modulating vector populations or pathogen survival in the environment.

## Background

Interactions between domestic and wild ungulates represent a potential problem in epidemiology [1], but little is known about the role of roe deer (*Capreolus capreolus*) in some diseases of concern in livestock. The roe deer is a Eurasian wild cervid whose populations have been expanding during the last decades across Europe, both in density and in geographical range [2,3]. These demographic and geographic changes may increase the risk of acquiring new diseases through both increased contact rates with other species, and increased intra-specific contact and density-dependent impact on individual fitness at higher densities [4,5]. Expansion of roe deer may have an influence in the epidemiology of several infectious diseases potentially shared with other native wild ungulates, domestic ungulates, and even human beings [1].

In Europe, several serologic surveys have been carried out in order to investigate the sanitary status of roe deer in different countries and situations. These surveys have reported on Pestivirus and Herpesvirus, paratuberculosis and other bacterial diseases, and protozoa mainly including *Toxoplasma gondii *and *Neospora caninum*. However, only limited knowledge exists regarding diseases of roe deer from the Iberian Peninsula.

Infections with bovine viral diarrhea virus (BVDv), a Pestivirus, are widespread throughout the world. Although infection prevalence varies among surveys, the infection tends to be endemic in cattle, reaching a maximum level of 1% persistently infected (PI) and 60% antibody positive cattle. PI cattle are the main source for transmission of the virus [6]. In the US, white-tailed deer (*Odocoileus virginianus*) can get infected from cattle and give birth to PI fawns that may interfere with control programs [7]. In Europe, BVDv-like Pestivirus was isolated from two seronegative roe deer in Germany [8] and 12% seroprevalence was found in roe deer from Norway [9]. However, no Pestivirus seropositive roe deer were found in several recent surveys in Germany [10], Austria [11] and Italy [12,13]. Two studies carried out in the Spanish Pyrenees showed no antibody seroprevalence in 21 and 43 roe deer tested against these viruses [14,15].

Of the ruminant alpha-herpesviruses, Bovine Herpesvirus 1 (BHV-1) is the best characterized one and responsible for infectious bovine rhinotracheitis (IBR). However, other cross-serological related alpha-herpesviruses have been isolated from cervids [16]. Roe deer have been included in Bovine Herpesvirus serosurveys in Germany [10], Italy [12] and Norway [9], showing mean serum antibody prevalences of 10%, 0% and 3% respectively.

The possible role of wild ruminants, notably deer, in bluetongue epidemiology is a matter of increasing concern in Europe. Recent surveys reported low (≤ 5%) prevalence of bluetongue (BT) antibodies in roe deer from Spain [17], and from Belgium [18]. Despite this, the role of European wild ruminants in the epidemiology of BTV remains still unclear.

Regarding bacterial diseases, wild ruminants are susceptible to paratuberculosis, a disease caused by *Mycobacterium avium paratuberculosis *(MAP) [19,20]. Previous studies on MAP revealed an antibody seroprevalence up to 13% in roe deer from North-Western Italy and Norway [20,21]. In the Czech Republic, MAP infection was confirmed in 0.2% [19] and in Italy in 22% of roe deer examined [21]. A recent serosurvey on MAP antibodies, using the PPA3 antigen ELISA, revealed 3% prevalence in cattle from north-western Spain [22]. However, there is no information on paratuberculosis in roe deer from Spain.

In Spain, brucellosis in domestic ruminants is almost eradicated, and its prevalence in bovine (caused by *B. abortus*), caprine and ovine (caused by *B. melitensis*) herds has decreased from 1.3% and 12% in 2001 to 0.7% and 2.8% in 2007, respectively (http://rasve.mapa.es, last access 16/04/2010). It is believed that wild ruminants are occasional victims of brucellosis "spill-over" from livestock, rather than true reservoirs [1,23-25].

Contact with other infectious diseases recently reported in European roe deer includes papillomavirus [26], Q fever [27], chlamydiophilosis [28] and anaplasmosis [29]. Finally, several studies reported on antibodies against *Toxoplasma gondii *[12,30-36] and against *Neospora caninum *[12,36,37].

The roe deer is a selective browser and less gregarious than other deer species [38,39], the pasture sharing and contact with domestic cattle is low and disease maintenance is less likely [40]. Therefore, we hypothesized that roe deer would not be an important species for wildlife disease surveillance [41]. In contrast, we expect a different scenario with diseases not associated with behavior, such as vector borne diseases. The aim of this serosurvey was determining the seroprevalence of antibodies against Pestivirus, Herpesvirus, Bluetongue virus, MAP, and *Brucella *sp. in roe deer from different areas of Spain in order to infer the role that this species can play in their epidemiology.

## Methods

### Animal sampling

**Figure 1:**
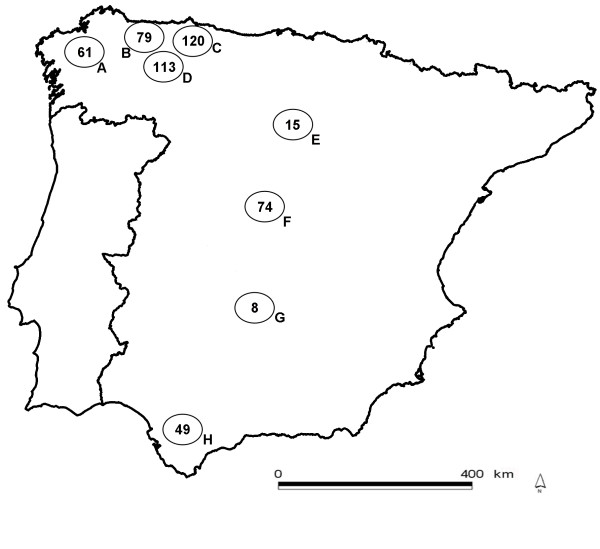
**In circles, the eight defined sampling populations with the number of sampled animals in each**. A, Coruña; B, Occidental Cantabrian Coast; C, Oriental Cantabrian Coast; D, Cantabrian mountains; E, Iberian mountains; F, Central mountains; G, Toledo mountains; H, Alcornocales.

Our study area was the Iberian Peninsula in south-western Europe. For the study, eight geographic sampled populations were defined (Figure 1). Sampling took place during an eight-year period, from hunting season 2000/2001 to 2008/2009, and was opportunistic and biased towards the main hunting season (summer). Blood from hunter-harvested animals was drawn from the heart or the thoracic cavity during field necropsies. Serum was obtained after centrifugation and stored at -20°C until analyzed. When possible, lymph nodes were collected and stored at -20°C for PCR testing. Sex and age were determined; the latter according to tooth eruption patterns [42]. Animals < 1 year of age were classified as calves, and those > 1 year of age as adults. Sex (n = 458) and age (n = 464) could be recorded for most animals. Due to hunting origin, the sample was biased towards adult males (n = 301).

### Laboratory techniques

Serologic tests and techniques employed are summarized in Table 1. In some cases, insufficient volume of sera did not allow testing for antibodies against all pathogens (Table 2). In order to verify the presence of pathogens, 49 roe deer sera from seropositive areas were tested by means of a sandwich ELISA that detects the BVD/MD/BD p125/p80 protein. Lymph node samples from five PPA3 ELISA seropositive roe deer were tested for the repetitive insertion sequence IS900 of *Mycobacterium avium paratuberculosis *by PCR (Adiavet paraTB, Adiagene, Saint-Brieuc, France; [43]).

**Table 1:** Serologic tests employed for serological assay of roe deer sera sampled

Agent (group)	Test	Antigen	Conjugate	Reference
BVDv/BDv (Pestivirus)	ELISA; SERELISA^® ^BVD/BD p80 Ab. Mono Blocking, Synbiotics; Lyon, France.	protein p80/125	None (blocking test)	[9]
	ELISA; SERELISA^® ^BVD/BD p80 Ag. Mono Indirect, Synbiotics; Lyon, France.	None	Goat Ab anti-rabbit Ig/peroxidase	[15]
IBRv (Herpesvirus)	ELISA; SERELISA^® ^IBR/IPV Ab. Mono Indirect Synbiotics; Lyon, France.	glycoprotein gB	MAb anti-bovine IgG/peroxidase	[12]
Bluetongue virus	Ingezim BTV Compac 2.0 12.BTV.K3^®^, Ingenasa; Madrid, Spain.	recombinant VP7	None (blocking test)	[58]
*M. avium paratuberculosis*	ELISA; In-house modified including positive controls.	PPA-3 (Allied Monitor, Fayette, MO, USA)	Protein-G/peroxidase	[43]
*Brucella *sp.	Rose Bengal agglutination test	Rose Bengal *Brucella *Antigen	None	[59]

**Table 2:** Serologic prevalence of selected infectious diseases in roe deer sampled, according to the eight sampling populations

Population	Sera analyzed	Pestivirus		Herpesvirus		BTV		MAP		*Brucella*	
		
		Sera*	Prevalence (%)	Sera*	Prevalence (%)	Sera*	Prevalence (%)	Sera*	Prevalence (%)	Sera*	Prevalence (%)
			95% CI		95% CI		95% CI		95% CI		95% CI
A	61	0/61	0	0/61	0	0/21	0	10/61	16.4	0/42	0
			(0-4.87)		(0-4.87)		(0-15.8)		(8.74-27.74)		(0-8.92)
B	79	1/79	1.3	0/79	0	0/27	0	8/79	10.1	0/37	0
			(0.07-6.75)		(0-4.75)		(0-12.38)		(4.76-18.84)		(0-9.05)
C	120	2/120	1.7	0/120	0	0/16	0	6/120	5	0/30	0
			(0.03-6.06)		(0-3.13)		(0-20.83)		(2.2-10.69)		(0-11.15)
D	113	2/113	1.8	0/113	0	0/42	0	11/113	9.7	0/26	0
			(0.03-6.44)		(0-3.32)		(0-8.92)		(5.16-16.7)		(0-12.85)
E	15	0/15	0	1/15	6.7	0/14	0	2/15	13.3	0/10	0
			(0-22.22)		(0.35-30.2)		(0-23.81)		(2.43-39.67)		(0-29.08)
F	74	3/74	4.1	0/71	0	0/41	0	7/74	9.46	0/19	0
			(1.12-11.29)		(0-4.79)		(0-8.17)		(4.53-18.72)		(0-17.65)
G	8	0/8	0	0/8	0	0/1	0	0/8	0	0/7	0
			(0-37.71)		(0-36.46)		(0-94.99)		(0-36.46)		(0-37.71)
H	49	0/49	0	0/49	0	0/10	0	4/49	8.2	0/30	0
			(0-7.65)		(0-7.65)		(0-29.08)		(2.84-19.18)		(0-11.15)
Total	519	8/519	1.5	1/516	0.2	0/172	0	48/519	9.2	0/201	0
			(0.05-3.04)		(0-1.1)		(0-2.18)		(7.01-12.11)		(0-1.87)

*positive sera/tested sera

### Statistics

We used Sterne's exact method to estimate prevalence confidence intervals. Prevalence comparisons among categories were done with homogeneity tests [44]. Data was analyzed using the SPSS statistical package, version 17.0 (SPSS Inc., Chicago, IL, USA).

## Results

The frequencies of antibody response against different pathogens are summarized in Table 2. No antibodies were detected against BT and *Brucella *sp. However, antibodies were detected against Pestivirus (1.5%), Herpesvirus (0.2%) and MAP (9.2%). MAP antibodies were detected in seven of the eight populations. Local prevalence was up to 16.4% in population A (Coruña; Table 2). Only one animal was seropositive for more than one pathogen (MAP and Pestivirus). When analyzing MAP seroprevalences by age and sex, no statistically significant differences were found (Fischer exact test, p > 0.05 in both cases). No MAP DNA was detected by PCR in the five PPA3 ELISA positive roe deer tested. Mean Pestivirus antigen prevalence in the seropositive areas was 16.3% (95% IC, 7.6-29.4).

## Discussion

This is the first large-scale survey on infectious disease agents in Iberian roe deer. Results reported herein confirm our initial hypothesis that roe deer display lower prevalence of antibodies against viral and bacterial diseases [12], as compared to other wild ruminants [17,27,43]. One possible explanation could be that differences in social behaviour between roe deer and other Iberian wild ruminants, such as red and fallow deer, would lead to fewer intra-specific contacts. Roe deer are seasonally territorial, solitary, and have smaller home ranges than red and fallow deer. Differences in feeding behaviour (roe deer are concentrate selectors and browsers) could lead to less indirect inter-specific contacts [18,38]. However, it must be taken into account that sampling was biased towards males, and may thus not represent the actual health situation of the roe deer population. Although serosurveys have proven to be a fundamental tool for disease surveillance, interpretation of antibody results in this study has to be approached with caution due to the lack of specific controls for roe deer [45]. Sensitivity and specificity for the different ELISA tests in roe deer were not determined in our study.

Pestivirus antibody prevalence was low, similar to results reported in other studies on European roe deer [11,13,14]. This suggests that roe deer have limited contact with common Pestivirus. In Spain, Pestivirus infection in domestic ruminants has frequently been reported, with prevalences reaching values up to 83% [14,46,47]. However, in our study a comparatively high antigen prevalence was found in the seropositive populations. This result contrasts with similar studies on wild ruminants, where antigen prevalence was always lower than antibody prevalence [48,49]. A possible explanation could be the presence of a new Pestivirus in this species not detectable by the antibody ELISA used [13]. New strains of Pestivirus have been described in the past in roe deer [50]. A second and more plausible explanation would be false positive results of the antigen ELISA due to unspecific cross-reactions. In order to clarify these findings, the samples will be further analyzed by PCR and serum neutralization.

The low antibody prevalence found against Herpesvirus was probably also a reflection of the relative isolation of roe deer from domestic animals. IBR is endemic among bovine livestock in Spain, with herd prevalence of 40-50% although vaccination programs are implemented (http://rasve.mapa.es, last access 16/06/2010).

Concerning BT, we expected some level of antibody detection based on prior data on wild ruminants from Spain [17,51] and Belgium [18], and because of being a vector borne disease, a priori less dependent on social behaviour and food habits. Few roe deer in our sample were harvested in BT areas (n = 65). The possible reasons for the marked difference in BT seropositivity between roe deer and sympatric red deer (*Cervus elaphus*) are open for further research [17,18].

Regarding brucellosis, results show that roe deer have no contact with *Brucella *sp. This confirms recent results from the Basque Country and Aragón, suggesting that roe deer constitute no reservoir host for livestock brucellosis in south-western Europe [25].

MAP is the pathogen with the highest antibody seroprevalence detected in our study. Serum antibody prevalence in the northern populations was lower than those reported in northern Spain in fallow deer (*Dama dama*) [52,53] and in red deer [43], but similar to that found for Cantabrian chamois (*Rupicapra pyrenaica parva*) [54]. Roe deer from Galicia (population A) displayed the highest seroprevalence. One possible explanation could be the eventual infection of wild ruminants feeding on pastures irrigated with liquid manure from infected dairy cattle [55], combined with the prolonged environmental survival of mycobacteria at high humidity and limited sunshine [56]. Population A inhabits a humid region with high percentage of dairy cattle and frequent use of liquid manure in contrast to the rest of the sampled areas where dairy cattle and use of their manure is much less important. The detection of MAP antibodies in samples from 7 of 8 roe deer populations throughout Spain suggests that contact with MAP may be widespread in this wild ruminant. However, all 5 PCR tested seropositive roe deer showed to be negative for MAP DNA. Negative results in this low number of PCR-tested animals can not rule out some possible involvement of roe deer in the epidemiology of this disease, or at least some potential of the roe deer as an indicator of environmental contamination by MAP, as already suggested for toxoplasmosis [31]. If wild ruminants were able to excrete MAP in sufficient quantities, circulation of MAP in wildlife could eventually interfere with MAP eradication efforts in livestock [57].

**Figure 2:**
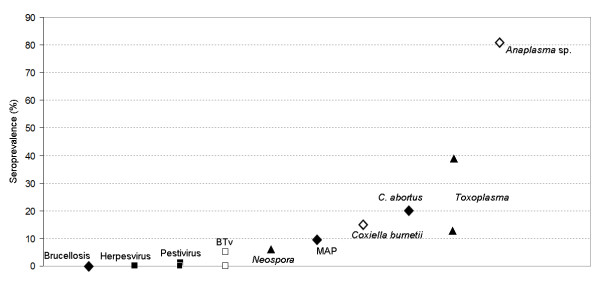
**Antibody prevalences found in 10 recent serosurveys of Spanish roe deer (2007-2010)**. Squares (■,□) indicate viruses, diamonds (♦,◊) are for bacterial diseases and triangles (▲) for parasites. White backgrounds indicate vector-borne diseases. References for Pestivirus, [14,15]; BTv, [17]; *Neospora*, [36,37]; *Coxiella burnetii*, [27]; *C. abortus*, [28], *Toxoplasma*, [31,36]; and *Anaplasma*, [29].

In order to put together recent information regarding roe deer serosurveys in Spain, Figure 2 shows the prevalence reported by agent. The figure suggests that this species has little contact with viral disease agents and *Brucella*, but seroprevalence increases when dealing with other bacterial and protozoan diseases. Excepting BT, vector-borne diseases have medium to high seroprevalence in roe deer. We suggest that antibody prevalences in roe deer are largely determined by environmental factors, potentially modulating vector populations or pathogen survival in the environment.

## Competing interests

The authors declare that they have no competing interests.

## Authors' contributions

AO, GP and MM, performed the field work and collected the samples of the study. MB and TC carried out the laboratory work. MB and CG analyzed the data and drafted the manuscript. All authors read and approved the final manuscript.
